# Reply to Wostyn, P. Could Young Cerebrospinal Fluid Combat Glaucoma? Comment on “Lee et al. Association between Optic Nerve Sheath Diameter and Lamina Cribrosa Morphology in Normal-Tension Glaucoma. *J. Clin. Med.* 2023, *12*, 360”

**DOI:** 10.3390/jcm12113784

**Published:** 2023-05-31

**Authors:** Seung Hyen Lee, Tae-Woo Kim, Eun Ji Lee, Hyunkyung Kil

**Affiliations:** 1 Department of Ophthalmology, Nowon Eulji Medical Center, Eulji University College of Medicine, Seoul 01830, Republic of Korea; 2 Department of Ophthalmology, Seoul National University Bundang Hospital, Seoul National University College of Medicine, Seongnam 13620, Republic of Korea; 3 Department of Ophthalmology, Bundang Jesaeng General Hospital, Seongnam 13590, Republic of Korea

We are pleased to see that Peter Wostyn has contributed a Comment: “Could Young Cerebrospinal Fluid Combat Glaucoma?” [1] in response to our paper published in the *Journal of Clinical Medicine*.

The author proposed a treatment strategy to control intracranial pressure (ICP) based on our findings that lower cerebrospinal fluid (CSF) pressure, which can be expected with a small optic nerve sheath diameter (ONSD), may be involved in the pathogenesis of normal-tension glaucoma (NTG) [2]. In particular, the author introduced an approach to glaucoma treatment by changing the CSF composition, assuming that not only CSF pressure but also altered CSF composition affects the pathophysiology of NTG. 

Recent studies have suggested that changes in the composition of CSF may be involved in the pathogenesis of NTG. Wostyn et al. proposed an alternative pathogenesis of NTG in which CSF circulatory failure results in reduced neurotoxin clearance along the optic nerve [3]. Additionally, our group reported that increased T-tau protein in CSF is associated with thinner lamina cribrosa (LC) thickness [4]. This supports the common pathophysiology of glaucoma and Alzheimer’s disease as neurodegenerative diseases [5,6,7]. Considering all of these, the altered composition of CSF may have a role in the pathophysiology of glaucoma. However, the specific differences in CSF composition in NTG patients and the specific role of these proteins in the development of NTG are still the subject of much debate. 

Our study only indirectly speculated that a small ONSD was associated with a low CSF pressure [2] and did not prove it by measuring the actual CSF pressure. Moreover, it is beyond the scope of our study whether a small ONSD is associated with differing CSF composition or resulting in different CSF flow dynamics. Therefore, the result of our study cannot be used as supportive evidence for the potential benefit of refreshing CSF composition in NTG patients. 

In conclusion, modifying the composition of CSF to treat glaucoma is an innovative and intriguing idea that warrants further investigation. However, it is questionable whether the small ONSD in NTG patients can justify such treatment. Further research is required to enhance insight into the role of CSF pressure and composition in the pathogenesis of NTG.
